# Arthropod Bite Mastitis as a Mimicker of Breast Cancer

**DOI:** 10.7759/cureus.28541

**Published:** 2022-08-29

**Authors:** Inna Robrahn, Santo Maimone, Mark A Edgar

**Affiliations:** 1 Radiology, Mayo Clinic, Jacksonville, USA; 2 Laboratory Medicine and Pathology, Mayo Clinic, Jacksonville, USA

**Keywords:** mri, ultrasound, mammogram, breast cancer, breast imaging, biopsy, mastitis, breast

## Abstract

Arthropod bite mastitis is rarely encountered in imaging practices but can occur in all regions of the world. Diagnosis is often challenging as the offending agent is rarely identified. While most manifestations are self-limited, severe presentations can mimic malignant processes such as Paget’s disease and inflammatory breast cancer (IBC). This case demonstrates the diagnostic challenges sometimes encountered with arthropod bite mastitis as well as imaging findings both prior to and after interventions.

## Introduction

While insect and arachnid bites occur worldwide, mastitis resulting from these arthropod bites remains a rare clinical entity with few case reports described [1-3]. Arthropod bite symptoms are frequently self-limiting without any adverse outcome, but in rare cases, they may progress and cause morbidity. The clinical presentation of arthropod bites varies from small clusters of erythematous papules or ecchymoses to large areas of deep induration and ulceration [4]. Histology typically demonstrates dermal edema, as well as a mixed inflammatory infiltrate composed of lymphocytes, histiocytes, eosinophils, and sometimes neutrophils [4]. In severe cases, this has the potential to mimic Paget’s disease or inflammatory breast cancer (IBC), both clinically and on imaging. This case report exemplifies the diagnostic challenges that can occur with arthropod bite mastitis.

## Case presentation

A 56-year-old woman presented with a three-month history of a right breast erythematous macular rash with scaling, which persisted despite the application of a topical antibiotic. The patient reported no antecedent trauma as well as no associated drainage, fever, or chills. During this time, she worked in a grocery store and began handling boxes of produce. At times, she noted occasional spiders and insects around the produce containers.

**Figure 1:**
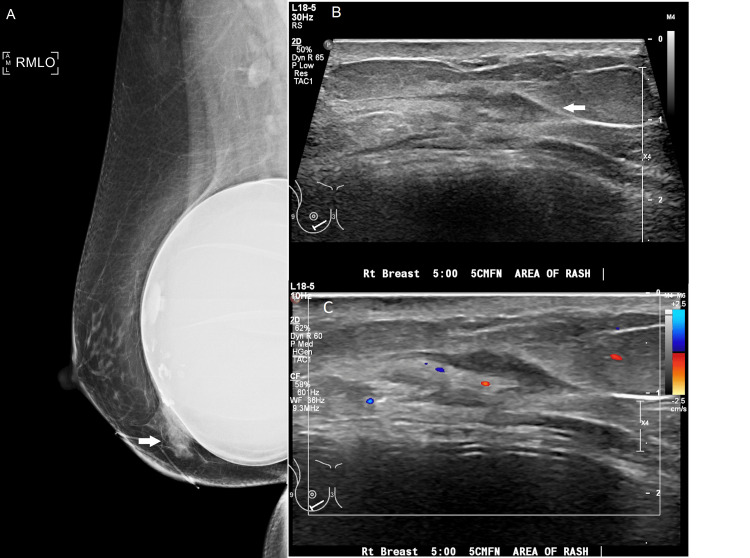
(A) Initial mammogram upon presentation showing an asymmetry (arrow) inferiorly with adjacent focal skin thickening. (B) Grayscale ultrasound image demonstrating nonspecific hypoechoic tissue (arrow) at the site of asymmetry on mammogram. (C) Color Doppler ultrasound image showing normal vascularity throughout the area of concern.

**Figure 2:**
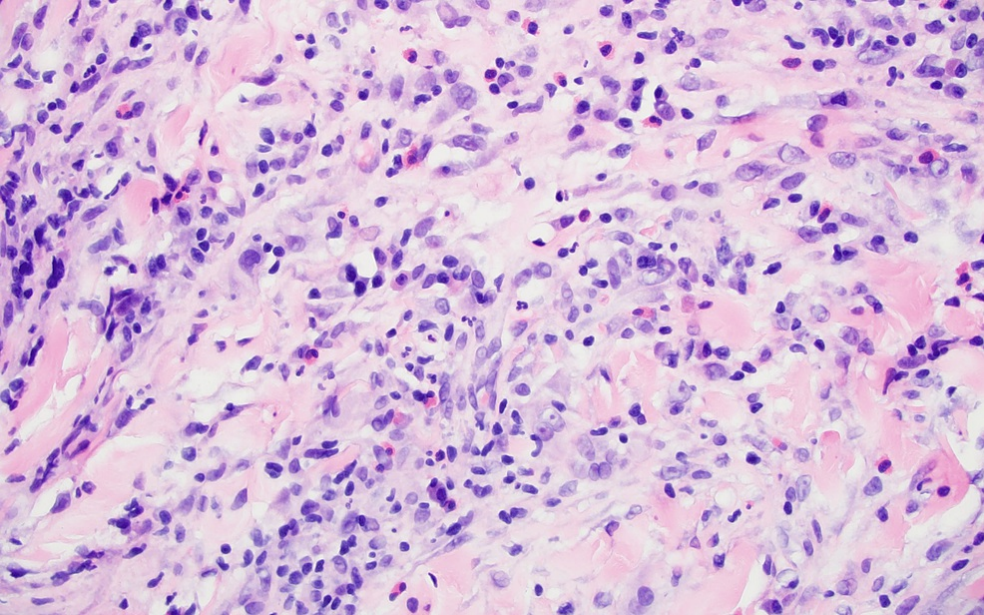
Hematoxylin and eosin stain (magnification: 400x) demonstrating polymorphous inflammatory cell infiltrate in the superficial dermis featuring lymphocytes, histiocytes, eosinophils, and neutrophils, typical of arthropod bite reactions.

Initial diagnostic mammography demonstrated an asymmetry with architectural distortion and skin thickening in the inferior right breast, with associated non-specific hypoechoic tissue on targeted ultrasound (Figure 1). Punch biopsy of the skin was recommended due to the focally suspicious appearance on clinical exam, safer access for tissue sampling, and to exclude Paget’s disease and inflammatory breast cancer. The punch biopsy hematoxylin and eosin stain showed crusted epidermal erosion with an underlying dermal hypersensitivity response, consistent with an arthropod bite (Figure 2). The biopsy result strengthened initial suspicions that a bite might have occurred when handling produce at work, although the specific offending arthropod was never identified. A prescribed course of topical steroid applications did not improve the patient’s symptoms.

**Figure 3:**
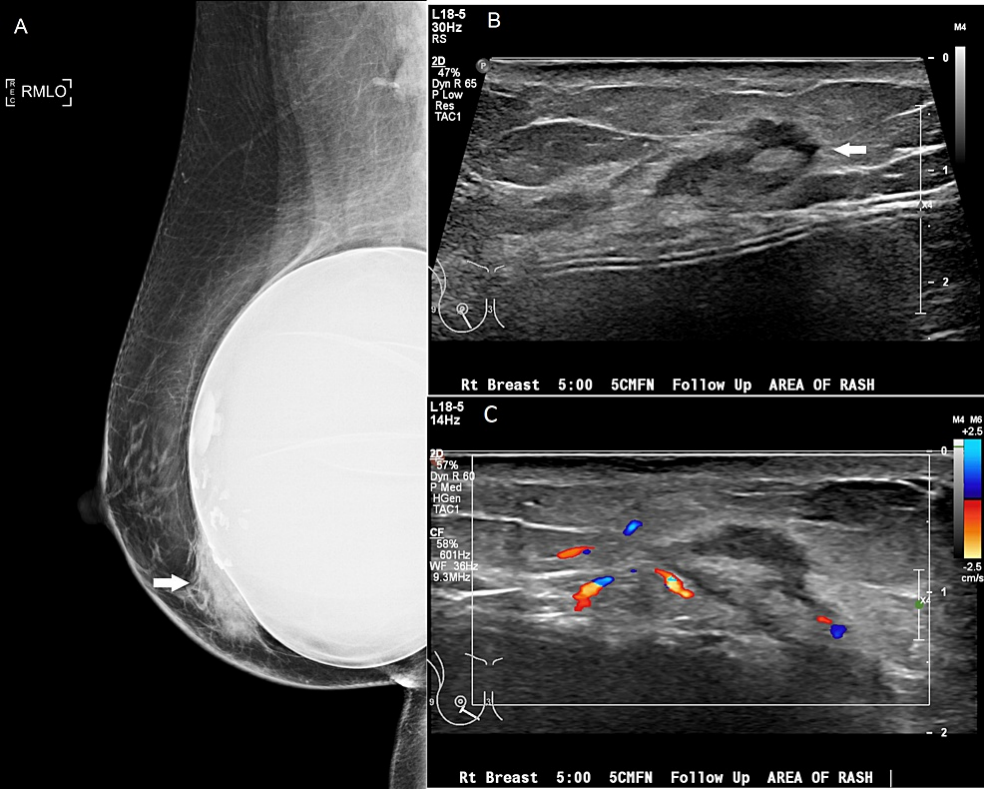
(A) Follow-up mammogram demonstrating an increase in size of the asymmetry (arrow) as well as an increase in skin thickening throughout the inferior breast (compare to Figure 1). (B) Grayscale ultrasound image demonstrating a mixed echogenicity mass (arrow), also increased in size and conspicuity (compare to Figure 1). (C) Color Doppler ultrasound image showing no suspicious vascularity associated with the lesion.

**Figure 4:**
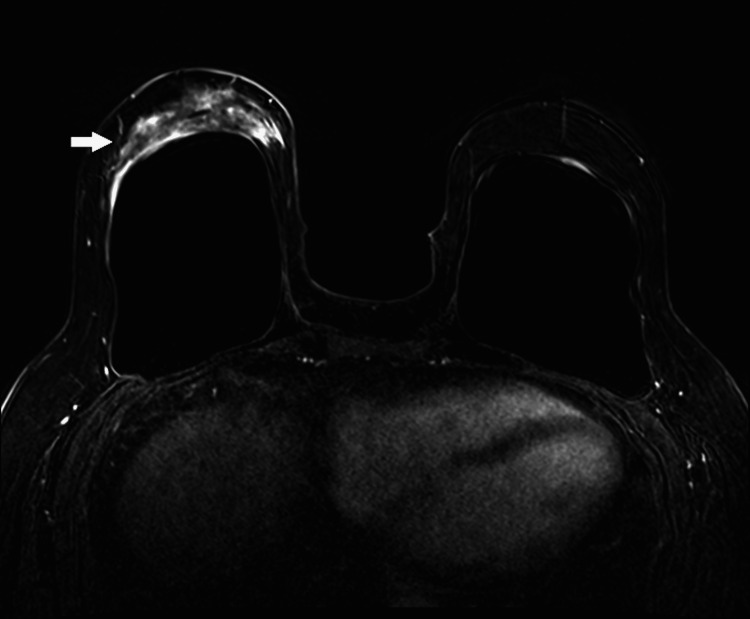
Post-contrast axial subtraction MRI image demonstrating heterogeneous regional non-mass enhancement in the inferior right breast (arrow), markedly asymmetric to the contralateral breast, which corresponded with the site of asymmetry on mammogram.

A short-term follow-up mammogram in four months demonstrated progressive skin thickening and an enlarging asymmetry in the inferior right breast, with a questionable underlying mass on targeted ultrasound (Figure 3). The findings were considered suspicious, so an ultrasound-guided core biopsy was recommended. Ultrasound-guided biopsy showed a hypersensitivity reaction. The patient was seen in the breast specialty clinic for continued clinical follow-up and a breast MRI was performed two months later. MRI demonstrated markedly asymmetric heterogeneous non-mass enhancement in the inferior right breast, which was suggested for excisional biopsy (Figure 4). Excisional biopsy showed dense fibrosis with acute and chronic inflammation without evidence of malignancy, with gross sectioning revealing scattered dense and focally indurated gray/white tissue without discrete mass. Since this last intervention, there has been no progression of symptoms over the last 15 months.

## Discussion

Although clinical presentations are rare, arthropod bite mastitis can occur worldwide and should be considered in breast imaging workups [2,4]. In many instances, making this diagnosis will be challenging as the offending agent is rarely visualized when the bite occurs and severe manifestations can mimic malignant processes [1,2]. Recognizing the potential clinical presentation can help guide management and limit extensive workups or unnecessary additional biopsies, as seen in this case.

No specific diagnostic imaging criteria are available for arthropod bites, although superficial edema and inflammation are expected [2]. Diagnosis is usually clinical, with the time of onset and visualization of potential offending agents providing significant assistance in determining etiology [1,2,4]. Clinical and imaging features of arthropod bite mastitis have the potential to mimic Paget’s disease of the breast and IBC, including skin erythema, skin edema/thickening, developing asymmetries on mammogram, nonspecific hypoechoic tissue on ultrasound, and non-mass enhancement on MRI [5,6]. In this case, a persistent rash with scaling, despite topical steroidal treatments, plus skin thickening and underlying asymmetry were indistinguishable from Paget’s disease without biopsy. Similarly, the progression of skin thickening with underlying asymmetry/distortion and abnormal enhancement raised concerns for IBC. A helpful distinguishing characteristic is the onset of symptoms, which is typically sudden and within 24 hours of the bite for arthropod bite mastitis, compared to a three-month interval for IBC [1,5]. However, if symptoms of arthropod bite mastitis persist and presentation is delayed, symptoms may be indistinguishable from the time course of IBC or Paget’s disease.

If arthropod bite mastitis is expected, clinical follow-up and possible short-interval follow-up imaging (three to six months) may be suggested to ensure a benign clinical exam or resolution of imaging findings. Specific treatments depend upon the arthropod responsible for the bite, with pharmacologic and nonpharmacologic approaches providing relief [7]. When clinical symptoms persist or progress, a biopsy may be needed to establish the correct diagnosis and rule out breast cancer. Awareness of arthropod bite mastitis can provide value in determining radiology/pathology concordance. This could help prevent unneeded subsequent biopsies, as interventions may exacerbate an inflammatory response and result in questioned progression on imaging and clinical exams, as noted in this case.

## Conclusions

Severe presentations of arthropod bite mastitis can mimic breast malignancy, including Paget’s disease and inflammatory breast cancer. Diagnosis is often challenging, and a biopsy may be needed to exclude an underlying malignant process. Awareness of this entity and reliable clinical information such as symptom onset and duration are key to preventing unnecessary additional interventions.
